# Prevalence of Depression and Anxiety Disorders Among Surgical Doctors in Public Hospitals in Makkah City, Saudi Arabia: An Analytical Cross-Sectional Study

**DOI:** 10.7759/cureus.33225

**Published:** 2023-01-01

**Authors:** Nahla Hariri, Nizar Bawahab, Elaf Banoon, Renad Abo Alshamat, Nami Almadani, Hamsa AlQashqri

**Affiliations:** 1 Department of Community Medicine and Healthcare of Pilgrims, Umm Al-Qura University, Makkah, SAU; 2 General Surgery, King Faisal Hospital, Makkah, SAU; 3 Emergency Department, King Abdulaziz Medical City, Jeddah, SAU; 4 General Surgery, Al Noor Specialist Hospital, Makkah, SAU; 5 Faculty of Medicine, Northern Border University, Arar, SAU

**Keywords:** surgeons, saudi arabia, anxeity, depression, prevalence rate

## Abstract

Background

Depression and anxiety are among the most prevalent illnesses worldwide. Although depression affects many individuals throughout their lives, physicians are at a heightened risk of developing the disorder due to their high-stress levels and enormous responsibilities. The study aimed to examine the prevalence of depression and anxiety disorders among surgical doctors in Makkah city hospitals as well as the risk factors.

Methods

Analytical cross-sectional research was performed at all public hospitals in Makkah city, Saudi Arabia. The Arabic version of the Hospital Anxiety and Depression Scale (HADS) was used as the screening tool.

Results

Of the 153 surgeons, 47.1% were Saudis, 81.7% were men, and 46.7% were residents. A personal history of anxiety or depression was apparent among 11.8% of the surgeons, while 4.6% had a family history of a mental disorder. Based on the HADS questionnaire responses, the prevalence rate of anxiety was 30.7%, and 27.5% of the surgeons had depression. Based on the univariate regression analysis, anxiety was significantly linked with being a participant in the Saudi Board program (p=0.010), working for more than nine hours (p=0.020), and having work-related stress (p=0.011 for moderate stress and p=0.001 for severe stress) as well as related to those who were rarely or never satisfied with their income (p=0.016 and p=0.047, respectively) and who was rarely satisfied with their career (p=0.019). Depression among surgeons was significantly linked with the age of 50 to 59 (p=0.023) as well as related to those who were usually satisfied with their career (p=0.022) and those with moderate work-related stress (p=0.016).

Conclusion

Psychological disorders such as depression and anxiety are prevalent among surgeons. They should be appropriately evaluated at regular intervals throughout life, especially during residency programs, to ensure physical and mental well-being, appropriate training exposure, and patient safety.

## Introduction

Depression and anxiety are among the most prevalent illnesses that affect an individual's personal and social life. Depression is a prevalent mental disorder, affecting an estimated 4% of the global population [1]. It is a multifaceted mask that many doctors unintentionally wear. As depression affects many individuals throughout their lives, physicians are at a heightened risk of developing the disorder due to their high-stress levels and enormous responsibilities. Numerous variables greatly influence the prevalence of depression among healthcare professionals, including smoking, a lack of leisure activity, and a lack of sleep [2]. In addition, symptoms have been linked to poor self-reported physical health [3], frequent workplace violence [4], long working hours, and frequent night shifts [5,6]. Surgeons have been reported to have elevated degrees of sadness and anxiety as a consequence of their rising workload and on-duty hours [6]. This has detrimental effects on both their physical and mental health as well as increases medical mistakes, which impacts patient safety and treatment quality [7-9].

Numerous studies of the incidence of depression and anxiety among doctors have been conducted worldwide. In Lahore, Pakistan, a study found that 34% of men and 24.8% of women had mild to moderate anxiety and depression, while 7.2% and 1.0% had severe anxiety and depression, as determined by the Standardized Hospital Anxiety and Depression Scale (HADS) [10]. Gu et al. investigated the incidence of depression among Southeast Nigerian resident doctors using a cross-sectional study with 300 resident and 150 non-resident physicians and the Mini-International Neuropsychiatric Interview technique. The findings revealed a significant frequency of depression among 17.3% of residents compared with 1.3% of non-resident physicians [11].

Long, irregular working hours, a stressful work atmosphere, and near-daily high-stakes procedures offer frequent challenges for surgical doctors [9]. Recent evidence suggests that such mental burdens might impact treatment success. In the United States, in an online cross-sectional survey of all The Accreditation Council for Graduate Medical Education (ACGME)-accredited general surgery programs, the rate of burnout among the 566 surgical residents were found to be 69%, driven equally by emotional stress and depersonalization. The researchers discovered a correlation between high stress and burnout among trainees and an increased rate of depression and suicidal thoughts [12]. The cross-sectional study by Shanafelt et al. using a sample size of 7905 surgeons who are American College of Surgeons members, found that 6.3% of the surgeons reported suicide ideation. Of these, 26% with recent suicide ideation had sought psychological help, while 60.1% refused to seek help [13].

Few studies have examined the frequency of depression and anxiety among physicians in Makkah city, Saudi Arabia. One cross-sectional study assessed the impact of excessive working hours on anxiety and depression among 258 doctors. Anxiety was prevalent among 39.5%, whereas depression was prevalent in 20.9%. The amount of sleep, exposure to injustice, and working more than 64 hours per week were significantly related to an increase in the risk of noticing greater levels of mental illness [14]. Another study was conducted in Makkah city among a sample of 100 doctors, 68% of whom were men, who worked in the emergency room of a hospital. The results demonstrated that depression and anxiety were prevalent among these emergency doctors (47%). Of these cases, 51% had a mild form, 40.4% had a moderate form, and 8.5% had a severe form [15]. Moreover, a cross-sectional epidemiological investigation of 90 primary care doctors in Makkah using a self-administered questionnaire reported three types of depression among these doctors; however, 28.9% showed mild depression, 18.9% moderate depression, and 10% severe depression. Further, more female doctors than men reported depressive symptoms [16].

Hence, numerous studies have found a high incidence of depression and anxiety across a variety of medical specializations. However, research on depression and anxiety among surgeons in Saudi Arabia is sparse. The purpose of this research is to identify the prevalence of depression and anxiety among surgeons at Makkah hospitals as well as the risk factors.

## Materials and methods

Study design

This study employs an analytical cross-sectional design to assess the prevalence of depression and anxiety among surgeons at all public hospitals in Makkah city, Saudi Arabia, as well as the risk factors.

Sampling procedure

All public hospitals in Makkah city were chosen for their surgical services. Data collection was scheduled to collect the surgeons' responses after finishing their morning sessions daily in each surgical department. We distributed data collectors in each surgical department at each hospital to ensure each department had the same chance to participate in the study. Simple random probability sampling was used to recruit the surgeons. The researchers gave each surgeon a copy of the questionnaire developed for this study. The introduction explained the goal of the research and emphasized that participation was optional. Each copy contained the HADS and demographic questions. Surgeons were instructed to put completed surveys in a box at the nursing station in each surgical department.

Study population

The study included surgeons in all surgical specialties (general surgery, cardiothoracic surgery, neurosurgery, plastic surgery, pediatric surgery, vascular surgery, orthopedics surgery, ears, nose, throat [ENT] surgery) and at all levels (service, residents, specialists, consultants) in all public hospitals in Makkah city, Saudi Arabia.

Sample size

With a population of 454 surgeons in Makkah hospitals and estimating the prevalence of depression to be 20% [14], therefore the sample size to be no less than 150 participants was required to gain a 95% confidence interval (i.e., that the obtained rate was within ±5%).

Measures

Sociodemographic questions were used to obtain variables such as age, gender, marital status, nationality, number of children, job title, workplace, participation in the Saudi Board program, type of specialty, number of years in surgery, working hours per day, number of on-calls per month, type of on-call, presence of post-call day off, career satisfaction, monthly income, income satisfaction, smoking, depression/anxiety diagnosis, family history of mental illnesses, tension at home, and stress at work. The HADS was used as a screening measure [17], which has been developed to determine the levels of anxiety and sadness as well as common somatic symptoms of illness (e.g., fatigue and insomnia). The questionnaire is scored on a range from 0 to 3 to determine a person's level of anxiety or depression. Items 1, 3, 5, 7, 9, 10, and 13 represent depression symptoms, and items 2, 4, 6, 8, 11, 12, and 14 represent anxiety symptoms. The grading is as follows: 0-7 - no depression or anxiety, 8-10 - borderline sadness or anxiety, and 11 or more - depression or anxiety cases. The questionnaire is presented in the appendix. The Cronbach's alpha values for the Arabic version of the HADS were 0.83 for the anxiety subscale and 0.77 for the depression subscale [18]. The inclusion criteria were if respondents were residents, specialists, or consultants, while physicians taking rotations from other specialties and interns in the surgical specialty were excluded.

Statistical analysis

Statistical analysis was carried out using RStudio (R version 4.1.1; R Foundation for Statistical Computing, Vienna, Austria). Frequencies and percentages were used to express the categorical variables, whereas the numerical scores of depression and anxiety were presented as medians and interquartile ranges [IQRs]). Univariate logistic regression analysis was used to assess the factors linked with depression and anxiety. The significantly associated factors were subsequently added into multivariate binary logistic regression models to investigate the independent risk factors for depression and anxiety. A p-value of <0.05 indicated statistical significance.

## Results

Sociodemographic characteristics

Initially, we received responses from 176 surgeons in the current study. However, we excluded the responses of 23 participants due to missing data on the primary outcomes (responses for the HADS questionnaire). Therefore, we analyzed the responses of 153 surgeons. Almost one-third of the surgeons were from 30 to 39 years old (34.6%), and fewer than half were Saudis (47.1%). The majority of the participants were men (81.7%), and more than half had a monthly income of 10,000 to 20,000 Saudi riyal (SAR; 52.6%). Married respondents represented 71.9% of the sample, and 66.7% had children. A personal history of anxiety or depression was apparent among 11.8% of the surgeons, while 4.6% had a family history of a mental disorder (Table 1).

**Table 1:** Sociodemographic characteristics of the participants SAR - Saudi riyal

Parameter	Category	n (%)
Age (years)	<30	39 (25.5%)
	30 to 39	53 (34.6%)
	40 to 49	42 (27.4%)
	50 to 59	18 (11.8%)
	≥60	1 (0.7%)
	Missing	0 (0%)
Sex	Male	125 (81.7%)
	Female	28 (18.3%)
	Missing	0 (0%)
Nationality	Saudi	72 (47.1%)
	Non-Saudi	81 (52.9%)
	Missing	0 (0%)
Marital status	Single	40 (26.1%)
	Married	110 (71.9%)
	Divorced	3 (2.0%)
	Widowed	0 (0.0%)
	Missing	0 (0%)
Monthly income (SAR)	<10,000	30 (19.6%)
	10,000 to 20,000	80 (52.3%)
	>20,000	42 (27.4%)
	Missing	1 (0.7%)
Have children	Yes	102 (66.7%)
	No	51 (33.3%)
	Missing	0 (0%)
Smoker	Yes	57 (37.3%)
	No	96 (62.7%)
	Missing	0 (0%)
History of depression or anxiety	Yes	18 (11.8%)
	No	135 (88.2%)
	Missing	0 (0%)
Family history of a mental disorder	Yes	7 (4.6%)
	No	146 (95.4%)
	Missing	0 (0%)

Occupational characteristics

**Figure 1:**
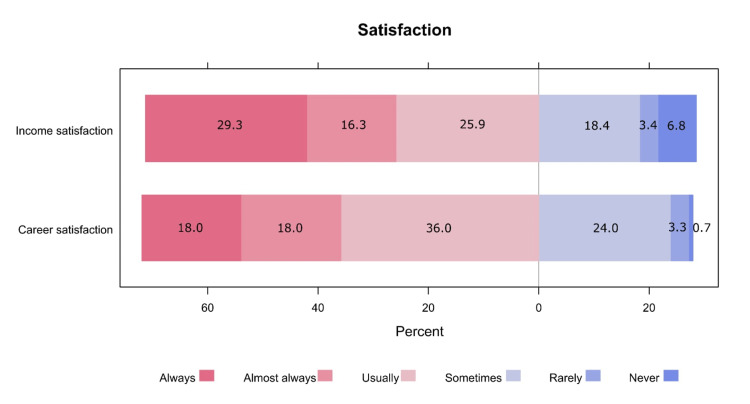
Participants' satisfaction with their income and career

Fewer than half of the respondents were residents (46.7%), and 28.9% of the sample had participated in the Saudi Board program. There were 39.3% of respondents with low experience (0 to 5 years), and the majority of the participants (74.2%) worked for eight to nine hours per day. Fewer than half of the participants had five to six on-calls per month, and 63.8% had in-house on-calls (the need to be promptly accessible at the hospital). Moderate to severe self-reported stress was attributed to home life by 42.7% of the participants and to work by 79.7% (see Table 2). More than one-quarter of the included surgeons were dissatisfied (responding as sometimes, rarely, or never) with their income (28.6%) and career (28.0%, see Figure 1).

**Table 2:** Occupational characteristics of the participants

Parameter	Category	n (%)
Job title	Resident	71 (46.7%)
	Specialist	46 (30.3%)
	Consultant	35 (23.0%)
	Missing	1 (0.7%)
Participation in the Saudi Board program	Yes	43 (28.1%)
	No	106 (69.3%)
	Missing	4 (2.6%)
Specialty in surgery	General surgery	70 (45.7%)
	Pediatric	10 (6.5%)
	Orthopedic surgery	24 (15.7%)
	Vascular	7 (4.6%)
	Urology	4 (2.6%)
	Thoracic	7 (4.6%)
	Colon and rectal	0 (0.0%)
	Otolaryngology	8 (5.2%)
	Neurological	9 (5.9%)
	Missing	14 (9.2%)
Number of practicing years in surgery	0 to 5	57 (37.3%)
	6 to 10	41 (26.8%)
	>10	47 (30.7%)
	Missing	8 (5.2%)
Working hours per day	8-9 hours	112 (73.2%)
	>9 hours	39 (25.5%)
	Missing	2 (1.3%)
Number of on-calls per month	≤4	48 (31.4%)
	5 to 6	64 (41.8%)
	>6	41 (26.8%)
	Missing	0 (0%)
Type of on-call	In-house	97 (63.4%)
	Home	39 (25.4%)
	Both	16 (10.5%)
	Missing	1 (0.7%)
Take post-call day off	Yes	79 (51.6%)
	No	74 (48.4%)
	Missing	0 (0%)
Home stress	Light	86 (56.2%)
	Moderate	57 (37.2%)
	Severe	7 (4.6%)
	Missing	3 (2.0%)
Work stress	Light	29 (19.0%)
	Moderate	84 (54.9%)
	Severe	30 (19.6%)
	Missing	10 (6.5%)

Description of anxiety and depression

**Figure 2:**
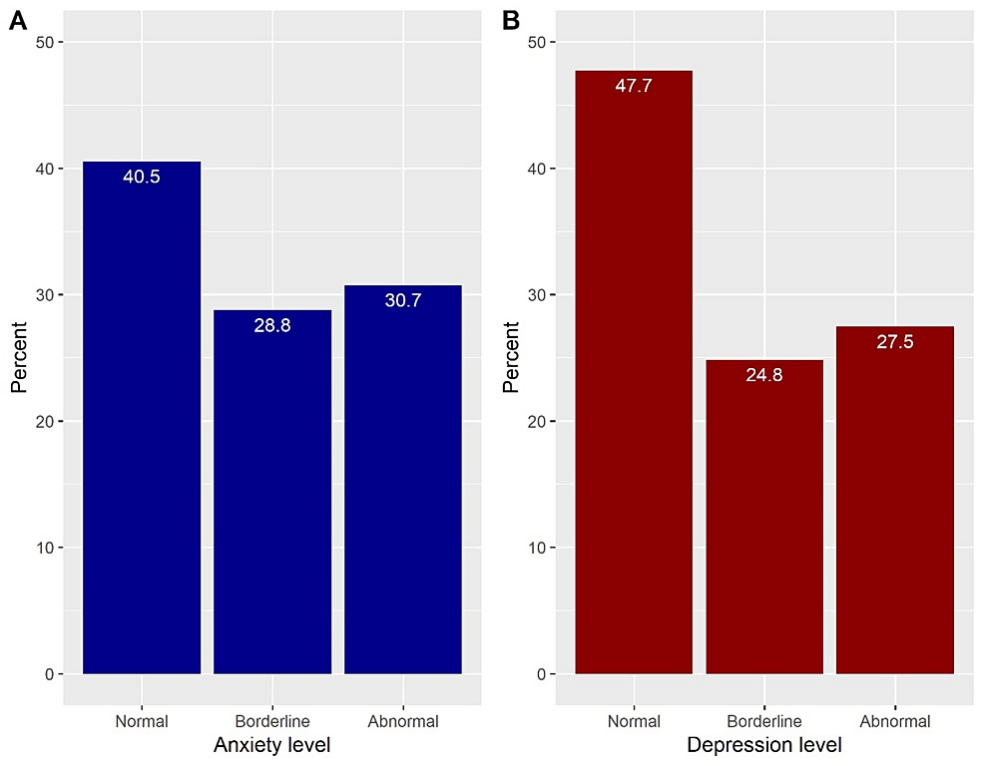
Levels of anxiety (A) and depression (B)

Based on the HADS questionnaire responses, the median anxiety score was 9 (IQR: 6-11), and the median depression score was 8 (IQR: 5-11). The prevalence rates of anxiety were 30.7%, and 28.8% of the participants had borderline anxiety (Figure 2A and Table 3). Additionally, 27.5% of the surgeons had depression, whereas borderline depression was prevalent among 24.8% (Figure 2B and Table 3).

**Table 3:** Descriptive statistics of anxiety and depression

Parameter	Anxiety	Depression
Numerical score (median, IQR)	9 (6-11)	8 (5-11)
Number of items	7	7
Cronbach’s alpha	0.698	0.694
Normal, n (%)	62 (40.5)	73 (47.7)
Borderline, n (%)	44 (28.8)	38 (24.8)
Case, n (%)	47 (30.7)	42 (27.5)

Factors associated with anxiety

The results of the univariate regression analysis showed that anxiety differed significantly based on the participants' age (p=0.036), nationality (p=0.040), marital status (p=0.001), having children (p=0.007), and being diagnosed with depression or anxiety (p=0.019, Table 4). Additionally, anxiety was significantly associated with being a participant in the Saudi Board program (p=0.010), working for >9 hours (p=0.020), and having work-related stress (p=0.011 for moderate stress and p=0.001 for severe stress), as well as related to those who were rarely or never satisfied with their income (p=0.016 and p=0.047, respectively) and who were rarely satisfied with their career (p=0.019). The incidence of anxiety was significantly lower among those who take home on-calls (p=0.017), specialists (p=0.009), and consultants (p=0.20, Table 5). However, upon adding the significantly associated variables into a multivariate model, we found that a higher level of work stress was the sole predictor of anxiety among the surgeons, including moderate (OR=12.3, 95% CI: 1.47-291, p=0.045) and severe stress (OR=15.6, 95% CI: 1.51-404, p=0.040, Table 6).

**Table 4:** Association between demographic characteristics and the presence of anxiety and depression OR - odds ratio; CI - confidence interval; NA - the record is non-available due to a small or zero frequency

Parameter	Category	Anxiety		Depression	
		OR (95% CI)	p	OR (95% CI)	p
Age	<30	-		-	
	30 to 39	0.61 (0.26 - 1.44)	0.26	2.83 (1.04 - 8.60)	0.05
	40 to 49	0.35 (0.13 - 0.91)	0.036	1.72 (0.57 - 5.57)	0.344
	50 to 59	0.37 (0.09 - 1.25)	0.127	4.40 (1.25 - 16.5)	0.023
	≥60	NA	0.987	NA	0.988
Sex	Male	-		-	
	Female	2.32 (0.99 - 5.40)	0.05	1.07 (0.41 - 2.59)	0.883
Nationality	Saudi	-		-	
	Non-Saudi	0.48 (0.24 - 0.96)	0.04	1.26 (0.62 - 2.61)	0.522
Marital status	Single	-		-	
	Married	0.28 (0.13 - 0.60)	0.001	1.03 (0.47 - 2.39)	0.934
	Divorced	NA	0.99	NA	0.991
Monthly income	<10,000	-		-	
	10,000 to 20,000	0.88 (0.37 - 2.16)	0.775	0.57 (0.24 - 1.39)	0.209
	>20,000	0.41 (0.14 - 1.17)	0.099	0.35 (0.12 - 1.01)	0.054
Have children	No	-		-	
	Yes	0.37 (0.18 - 0.77)	0.007	1.35 (0.63 - 3.02)	0.443
Smoker	No	-		-	
	Yes	1.07 (0.52 - 2.16)	0.859	0.79 (0.37 - 1.65)	0.538
History of depression or anxiety	No	-		-	
	Yes	3.31 (1.22 - 9.30)	0.019	1.37 (0.45 - 3.82)	0.553
Family history of mental disorders	No	-		-	
	Yes	3.19 (0.68 - 16.8)	0.139	1.06 (0.15 - 5.14)	0.946

**Table 5:** The association between occupational characteristics and the presence of anxiety and depression OR - odds ratio; CI - confidence interval; NA - the record is non-available due to a small or zero frequency

Parameter	Category	Anxiety		Depression	
		OR (95% CI)	p	OR (95% CI)	p
Job title	Resident	-		-	
	Specialist	0.31 (0.13 - 0.72)	0.009	0.71 (0.29 - 1.66)	0.438
	Consultant	0.32 (0.12 - 0.80)	0.02	1.33 (0.55 - 3.16)	0.519
Participation in the Saudi Board program	No	-		-	
	Yes	2.68 (1.27 - 5.67)	0.01	1.21 (0.54 - 2.61)	0.637
Specialty in surgery	General surgery	-		-	
	Pediatric	0.67 (0.10 - 2.98)	0.633	0.26 (0.01 - 1.51)	0.214
	Orthopedic surgery	2.27 (0.86 - 5.98)	0.094	0.61 (0.18 - 1.77)	0.389
	Vascular	NA	0.986	0.93 (0.13 - 4.72)	0.937
	Urology	0.89 (0.04 - 7.48)	0.925	7.00 (0.84 - 146)	0.1
	Thoracic	0.45 (0.02 - 2.86)	0.47	0.39 (0.02 - 2.47)	0.395
	Otolaryngology	2.68 (0.58 - 12.4)	0.192	0.33 (0.02 - 2.04)	0.318
	Neurological	0.77 (0.11 - 3.52)	0.754	1.17 (0.23 - 4.88)	0.838
Number of practicing years in surgery	0 to 5	-		-	
	6 to 10	0.61 (0.25 - 1.42)	0.26	1.75 (0.72 - 4.34)	0.218
	>10	0.45 (0.19 - 1.05)	0.069	1.29 (0.53 - 3.17)	0.57
Working hours per day	8-9 hours	-		-	
	>9 hours	2.45 (1.15 - 5.26)	0.02	1.97 (0.90 - 4.27)	0.088
Number of on-calls per month	≤4	-		-	
	5 to 6	0.73 (0.32 - 1.69)	0.465	0.97 (0.42 - 2.29)	0.951
	>6	1.41 (0.59 - 3.41)	0.444	1.11 (0.44 - 2.83)	0.819
Type of on-call	In-house	-		-	
	Home	0.31 (0.11 - 0.76)	0.017	0.78 (0.31 - 1.80)	0.57
	Both	0.77 (0.23 - 2.30)	0.652	1.18 (0.34 - 3.57)	0.779
Take post-call day off	No	-		-	
	Yes	1.24 (0.62 - 2.48)	0.544	0.80 (0.39 - 1.63)	0.541
Career satisfaction	Always	-		-	
	Almost always	0.77 (0.17 - 3.26)	0.715	0.31 (0.01 - 2.59)	0.321
	Usually	2.20 (0.75 - 7.42)	0.169	4.71 (1.41 - 21.6)	0.022
	Sometimes	2.49 (0.79 - 8.82)	0.132	4.00 (1.10 - 19.2)	0.05
	Rarely	17.6 (2.08 - 388)	0.019	32.0 (3.44 - 759)	0.007
	Never	NA	0.985	NA	0.99
Income satisfaction	Always	-		-	
	Almost always	1.15 (0.31 - 3.96)	0.825	0.66 (0.16 - 2.27)	0.526
	Usually	2.28 (0.83 - 6.53)	0.114	1.72 (0.65 - 4.64)	0.277
	Sometimes	2.19 (0.72 - 6.79)	0.167	1.16 (0.37 - 3.50)	0.8
	Rarely	17.5 (2.23 - 368)	0.016	4.95 (0.73 - 41.8)	0.103
	Never	4.37 (1.01 - 19.6)	0.047	2.20 (0.48 - 9.37)	0.286
Home stress	Light	-		-	
	Moderate	1.57 (0.76 - 3.27)	0.223	1.76 (0.83 - 3.75)	0.138
	Severe	3.88 (0.80 - 21.0)	0.091	2.64 (0.49 - 13.0)	0.228
Work stress	Light	-		-	
	Moderate	14.0 (2.76 - 256)	0.011	6.39 (1.74 - 41.5)	0.016
	Severe	32.0 (5.65 - 608)	0.001	4.91 (1.09 - 34.8)	0.059

**Table 6:** Results of the multivariate regression analysis of the risk factors for anxiety among the participants OR - odds ratio; CI - confidence interval; NA - the record is non-available due to a small or zero frequency

Parameter	Category	OR (95% CI)	p
Age	<30	-	
	30 to 39	1.47 (0.32 - 7.08)	0.621
	40 to 49	2.12 (0.25 - 19.5)	0.496
	50 to 59	2.47 (0.13 - 40.3)	0.527
	≥60	NA	0.998
Nationality	Saudi	-	
	Non-Saudi	0.27 (0.06 - 1.10)	0.074
Marital status	Single	—	
	Married	0.56 (0.05 - 4.83)	0.606
	Divorced	NA	0.995
Have children	No	-	
	Yes	0.93 (0.10 - 10.3)	0.946
Job title	Resident	-	
	Specialist	0.51 (0.08 - 2.98)	0.451
	Consultant	0.72 (0.06 - 8.85)	0.797
Participation in the Saudi Board program	No	-	
	Yes	0.79 (0.16 - 3.82)	0.77
Working hours per day	8–9 hours	-	
	>9 hours	1.08 (0.28 - 3.95)	0.904
Type of on-call	In-house	-	
	Home	0.50 (0.07 - 3.11)	0.47
	Both	1.02 (0.09 - 9.94)	0.986
Career satisfaction	Always	-	
	Almost always	4.31 (0.34 - 82.5)	0.287
	Usually	5.72 (0.84 - 68.3)	0.107
	Sometimes	3.21 (0.42 - 39.2)	0.297
	Rarely	NA	0.995
	Never	NA	0.998
Income satisfaction	Always	-	
	Almost always	0.84 (0.10 - 6.37)	0.868
	Usually	1.42 (0.32 - 6.66)	0.649
	Sometimes	1.23 (0.23 - 6.99)	0.811
	Rarely	NA	0.995
	Never	2.85 (0.27 - 30.0)	0.374
History of depression or anxiety	No	-	
	Yes	5.21 (0.72 - 46.1)	0.113
Work stress	Light	-	
	Moderate	12.3 (1.47 - 291)	0.045
	Severe	15.6 (1.51 - 404)	0.04

Factors associated with depression

Depression among the surgeons was significantly associated with the age of 50 to 59 (p=0.023), as well as with those who were usually satisfied with their career (p=0.022) and those with moderate work-related stress (p=0.016, Table 4 and Table 5). Nevertheless, advanced age (50 to 59 years) was independently associated with depression (OR=6.52, 95% CI: 1.26-37.50, p=0.028, see Table 7).

**Table 7:** Results of the multivariate regression analysis of the risk factors for depression among the participants OR - odds ratio; CI - confidence interval; NA - the record is non-available due to a small or zero frequency

Parameter	Category	OR (95% CI)	p-value
Age	<30	-	
	30 to 39	3.17 (1.02 - 11.3)	0.057
	40 to 49	1.42 (0.40 - 5.56)	0.594
	50 to 59	6.52 (1.26 - 37.5)	0.028
	≥60	NA	0.993
Career satisfaction	Always	-	
	Almost always	0.63 (0.03 - 6.03)	0.713
	Usually	4.53 (0.97 - 22.9)	0.059
	Sometimes	3.75 (0.93 - 19.7)	0.081
	Rarely	17.1 (1.05 - 539)	0.057
	Never	NA	0.99
Work stress	Light	-	
	Moderate	3.85 (0.85 - 27.7)	0.112
	Severe	2.83 (0.49 - 22.9)	0.27

## Discussion

A sizeable proportion of individuals report experiencing anxiety and/or depression at some point in their life. The majority are unwilling to undertake professional screening or therapy. Anxiety is described as a condition characterized by distress, fear, or intense worry, which may result in unpleasant mental and somatic symptoms. Depression is characterized by poor mood and reluctance to engage in regular activities. Anxious or depressed individuals are susceptible to poor mental and physical illness as well as the development of personality disorders. Doctors also experience mental illnesses [8]. A physician with a mental illness cannot provide services that are compatible with those of their mentally well colleagues. Doctors with anxiety or depression are often ineffective at work. The prevalence of mental illnesses, including anxiety and depression, substance abuse, and suicide, has never been thoroughly studied by medical professionals. In particular, long, irregular working hours, a stressful work atmosphere, and near-daily high-stakes procedures offer frequent challenges to surgical doctors. This research aimed to identify the prevalence rate of depression and anxiety disorders among surgical doctors in Makkah city and its related risk factors using the HADS.

In this study, we found that 46.7% of the sample were residents, and 28.9% had participated in the Saudi Board program. Moreover, 39.3% of the respondents had little experience (0 to 5 years), and the majority (74.2%) were working for eight to nine hours per day. Further, 41.8% of the participants had five to six on-calls per month, and 63.8% had in-house on-calls. Moderate to severe self-reported stress was attributed to home life by 42.7% of the participants and work life by 79.7%. More than one-quarter of the included surgeons were dissatisfied (responding as sometimes, rarely, or never) with their income (28.6%) and career (28.0%).

Based on the HADS questionnaire responses, this study reported the prevalence of anxiety as 30.7%, with 28.8% of the participants showing borderline anxiety. Additionally, 27.5 % of the surgeons had depression, with borderline depression prevalent among 24.8%. Previous studies have revealed similar results. For example, in Brazil, a cross-sectional study conducted in 2021 using the same screening tool found that 36.5% and 23.1 % of the 75 sampled surgeons had anxiety and depression, respectively [19]. Other studies have shown higher prevalence rates, as they have used different screening tools. For example, a study conducted in Kuwait found that the percentage of depression among surgeons was 55.3% using a Patient Health Questionnaire-9 (PHQ-9) score of 10 or above [20]. Another study undertaken in North Carolina reported that 39% of surgical doctors had depression using PHQ-9 [21].

According to the findings of the univariate regression analysis, this study found that anxiety differed significantly based on marital status (p=0.001) and having children (p=0.007). This is similar to the findings of research conducted among surgeons in Brazil, which revealed that marital status was highly linked to anxiety disorders [19]. This study also found that being diagnosed with depression or anxiety (p=0.019) increased the susceptibility to having anxiety. This finding supports an Australian study, which found that 18% of medical students and 21% of physicians diagnosed with depression have previously been diagnosed with depression [2].

Additionally, anxiety was considerably linked with being a participant in the Saudi Board program (p=0.010), working for more than nine hours (p=0.020), and having work-related stress (p=0.011 for moderate stress and p=0.001 for severe stress). These results are in line with earlier findings among depressed doctors. Research carried out in China found that single status (OR=1.13), a long employment history(OR=1.19), shift work (OR=1.91), and violence (OR=4.94) are factors that lead emergency physicians to be more susceptible to depression [22]. Another study conducted in Oman revealed that those who have six or more hospital on-calls per month are 2.65 times more prone to develop anxiety disorders than those who receive five or fewer on-calls per month [23]. This may be the result of the increased hours of study in preparation for upcoming professional tests and/or increased job pressure. As part of their training, resident physicians are continually exposed to an increasing workload and a continuous need to study.

Additionally, we found that advanced age (50 to 59 years) was independently associated with having depression (OR=6.52, 95% CI: 1.26-37.50, p=0.028). This is an important finding, as depression is a crucial concern for the elderly and those who care for the old. As the aged population grows, it is anticipated that the rate of depressed seniors could increase. Older doctors are not immune to depression. According to our research, interventions aimed at preventing, identifying, and treating surgeon depression and anxiety disorders are essential for preserving the well-being of the surgical profession.

Limitations

This was an analytical cross-sectional study design. Because independent and dependent variables are evaluated concurrently, the main drawback of the analytical cross-sectional research design is that there is often no proof of a temporal link between exposure and outcome. The research cannot be generalized since not all surgical doctors could be screened. Selection bias was unavoidable due to the low response rate from specialists and consultants.

In Makkah public hospitals, there has never been a study done like this making this work unique. A surprisingly high frequency of anxiety disorders and depression among surgeons was shown by the research, which produced significant data. Additional investigation and analysis of the risk factors might provide more fruitful findings.

## Conclusions

The high rate of depression and anxiety disorders reported among the surgical physicians in this study should motivate conducting more research on this topic. It would be easier to uncover the causes of depression and anxiety disorders, which contribute to physician turnover, if more robust research methods were used. To prevent health problems and reduce the likelihood of patient neglect, they should undergo appropriate screenings for depression and anxiety at regular intervals throughout their lives. Moreover, all medical facilities and programs must implement early prevention and management promptly.

## Appendices

Dear Surgeon,

This study is conducted by a research team and the research team leader is Dr Nahla Hariri (Assistant Professor, Department of Community Medicine and Pilgrim Health Care, Faculty of Medicine at Umm Al-Qura University).

The aim of the study is to find out the rates of Depression and Generalized Anxiety Disorder and related risk factors among Surgeons in Makkah city, Saudi Arabia.

Participation in this study is completely voluntary.

Please drop your completed questionnaire in the study box at reception of the nurses’ station in your department.  Please do not write your name on the form as your responses will be anonymous.

Please complete the following questionnaire, which will take about 5 minutes.

If you do not wish to participate in the study, you may leave now without any effect.

Thank you very much for your participation.

If you have any questions, please contact Dr Nahla Hariri (.sa)

Do you agree to participate?

o  Yes

o  No

1. Your age:

o <30  

o 30-39   

o 40-49    

o 50-59   

o ≥60

2. Gender:  

o Male              

o Female

3. Nationality:

o Saudi   

o Non-Saudi

4. Marital status:

o Single  

o Married  

o Divorced  

o Widowed

5. Do you have children:

o Yes   

o No

6. Job title:

o Resident

o Specialist 

o Consultant

7. Are you joining the Saudi Board program?

o Yes         

o No

8. Specialty in surgery:

o General surgery  

o Pediatric  

o Orthopaedic surgery   

o Vascular  

o Urology 

o Thoracic   

o Colon and rectal 

o Otolaryngology      

o Neurological  

o Plastic and maxillofacial surgery  

o Ophthalmic surgery

9. Number of practicing years in surgery?

o 0-5 

o 6-10  

o >10

10. Work hours per day:

o 8-9 hours      

o more than 9 hours  

11. Number of on-call per month:

o 4 or less   

o 5-6  

o more than 6

12. What type of on-call do you take?

o in-house 

o Home call 

o both

13. Do you take post-call day off?

o Yes       

o No

14. Career satisfaction:

o Always  

o Almost always 

o Usually 

o Sometimes

o Rarely 

o Never 

15. Monthly income:

o <10.000SR 

o 10.000-20.000SR  

o >20.000SR

16. Income satisfaction:

o Always  

o Almost always 

o Usually 

o Sometimes

o Rarely 

o Never

17. Do you smoke:

o Yes   

o No

18. Have you ever been diagnosed with depression or anxiety?

o Yes       

o No

19. Have you had a family history of mental disorders?

o Yes       

o No

20. Home stress:

o Light  

o Moderate 

o Severe

If your answer is moderate or severe, please specify the kind of home stress:

____________________________________________________________________

21.  Work stress:

o Light   

o Moderate 

o Severe

If your answer is moderate or severe, please specify the kind of work stress:

____________________________________________________________________

**Table 8:** Hospital Anxiety and Depression Scale (HADS) Tick the box beside the reply that is closest to how you have been feeling in the past week. D - depression; A - anxiety

D	A		D	A	
		I feel tense or 'wound up'			I feel as if I am slowed down
	3	Most of the time	3		Nearly all the time
	2	A lot of the time	2		Very often
	1	From time to time, occasionally	1		Sometimes
	0	Not at all	0		Not at all
		I still enjoy the things I used to enjoy			I get a sort of frightened feeling like 'butterflies' in the stomach
0		Definitely as much		0	Not at all
1		Not quite so much		1	Occasionally
2		Only a little		2	Quite often
3		Hardly at all		3	Very often
		I get a sort of frightened feeling as if something awful is about to happen			I have lost interest in my appearance
	3	Very definitely and quite badly	3		Definitely
	2	Yes, but not too badly	2		I don't take as much care as I should
	1	A little, but it doesn't worry me	1		I may not take quite as much care
	0	Not at all	0		I take just as much care as ever
		I can laugh and see the funny side of things			I feel restless as I have to be on the move
0		As much as I always could		3	Very much indeed
1		Not quite so much now		2	Quite a lot
2		Definitely not so much now		1	Not very much
3		Not at all		0	Not at all
		Worrying thoughts go through my mind			I look forward with enjoyment to things
	3	A great deal of the time	0		As much as I ever did
	2	A lot of the time	1		Rather less than I used to
	1	From time to time, but not too often	2		Definitely less than I used to
	0	Only occasionally	3		Hardly at all
		I feel cheerful			I get sudden feelings of panic
3		Not at all		3	Very often indeed
2		Not often		2	Quite a lot
1		Sometimes		1	Not very often
0		Most of the time		0	Not at all
		I can sit at ease and feel relaxed			I can enjoy a good book or radio or TV program
	0	Definitely	0		Often
	1	Usually	1		Sometimes
	2	Not often	2		Not often
	3	Not at all	3		Very seldom
